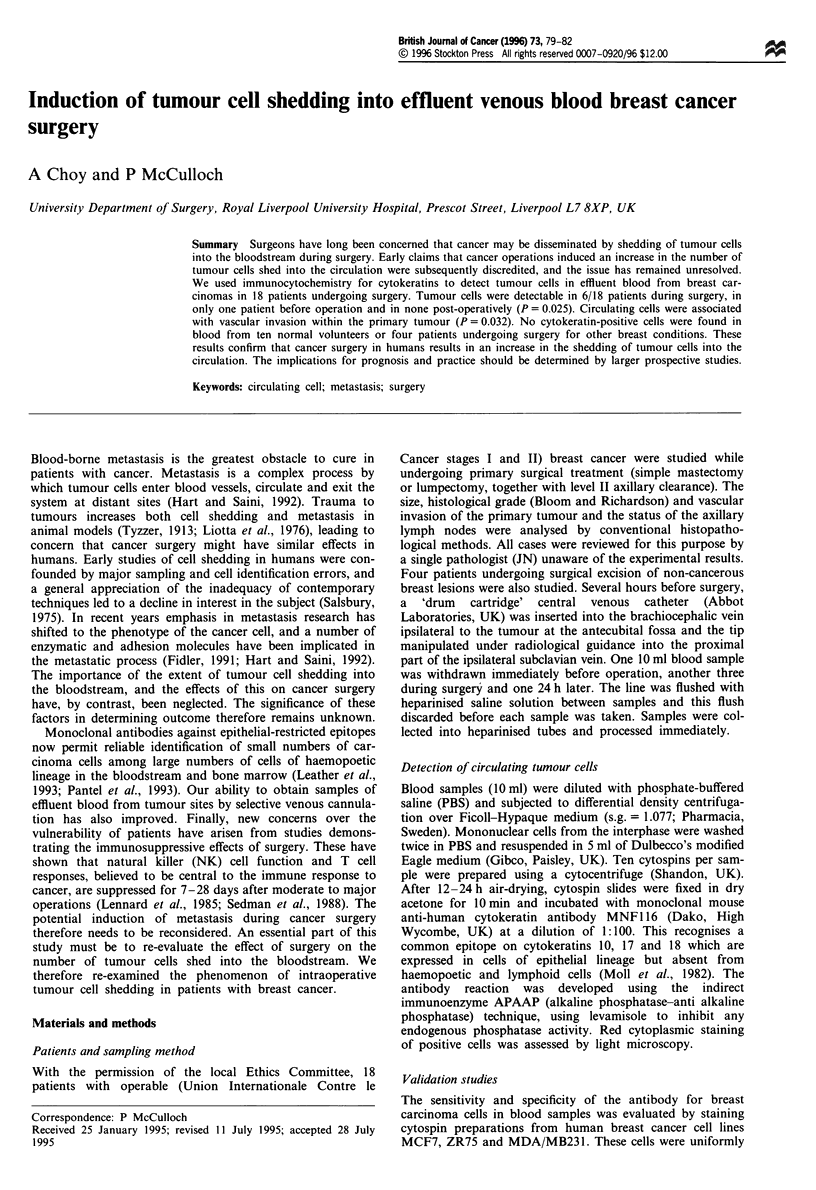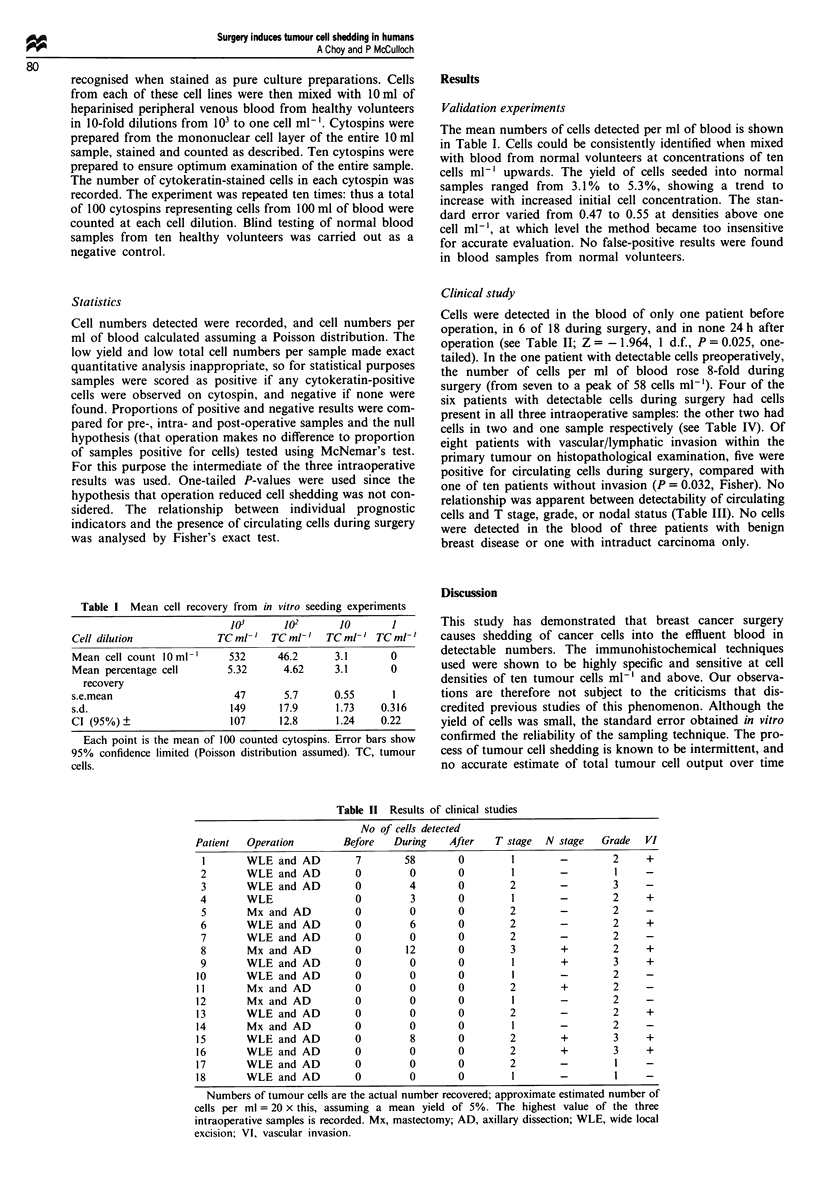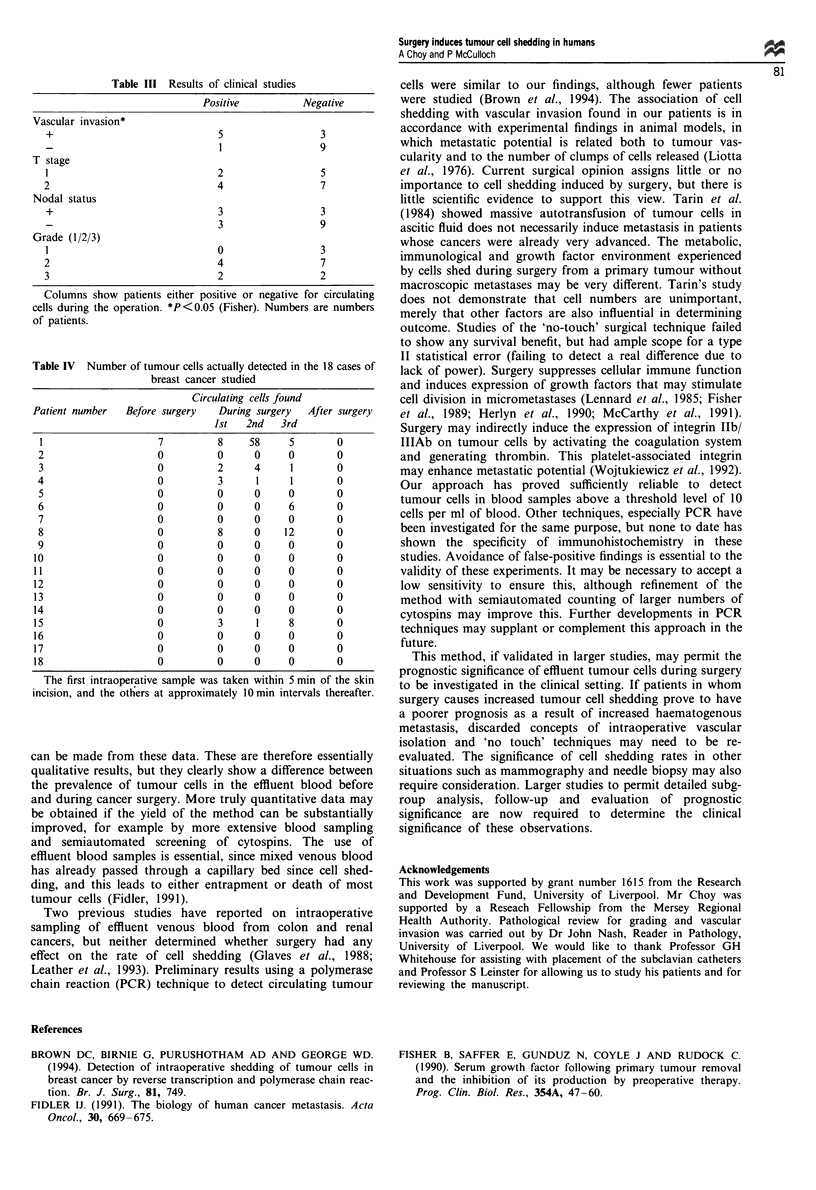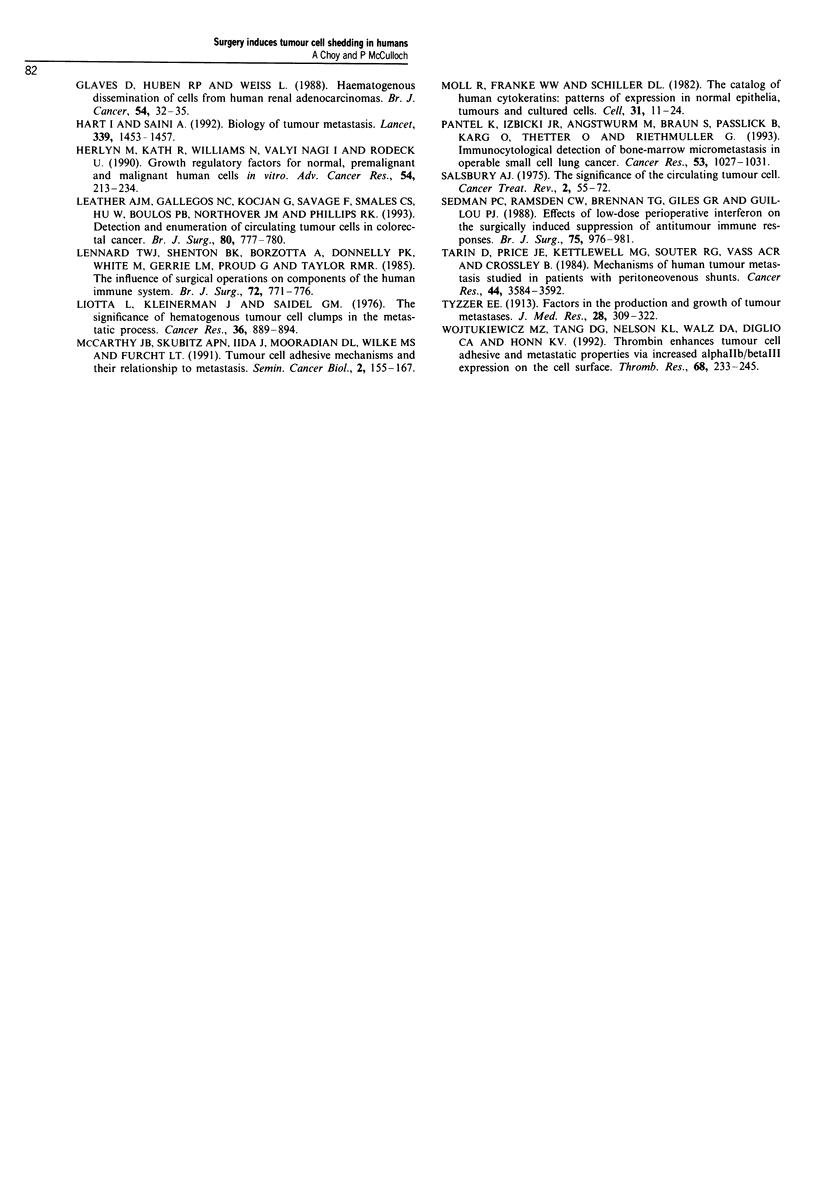# Induction of tumour cell shedding into effluent venous blood breast cancer surgery.

**DOI:** 10.1038/bjc.1996.14

**Published:** 1996-01

**Authors:** A. Choy, P. McCulloch

**Affiliations:** University Department of Surgery, Royal Liverpool University Hospital, UK.

## Abstract

Surgeons have long been concerned that cancer may be disseminated by shedding of tumour cells into the bloodstream during surgery. Early claims that cancer operations induced an increase in the number of tumour cells shed into the circulation were subsequently discredited, and the issue has remained unresolved. We used immunocytochemistry for cytokeratins to detect tumour cells in effluent blood from breast carcinomas in 18 patients undergoing surgery. Tumour cells were detectable in 6/18 patients during surgery, in only one patient before operation and in none post-operatively (P = 0.025). Circulating cells were associated with vascular invasion within the primary tumour (P = 0.032). No cytokeratin-positive cells were found in blood from ten normal volunteers or four patients undergoing surgery for other breast conditions. These results confirm that cancer surgery in humans results in an increase in the shedding of tumour cells into the circulation. The implications for prognosis and practice should be determined by larger prospective studies.


British Journal of Cancer (1996) 73, 79-82
© 1996 Stockton Press All rights reserved 0007-0920/96 $12.00

### Induction of tumour cell shedding into effluent venous blood breast cancer surgery

A Choy and P McCulloch

*University Department of Surgery, Royal Liverpool University Hospital, Prescot Street, Liverpool L7 8XP, UK*

**Summary** Surgeons have long been concerned that cancer may be disseminated by shedding of tumour cells into the bloodstream during surgery. Early claims that cancer operations induced an increase in the number of tumour cells shed into the circulation were subsequently discredited, and the issue has remained unresolved. We used immunocytochemistry for cytokeratins to detect tumour cells in effluent blood from breast carcinomas in 18 patients undergoing surgery. Tumour cells were detectable in 6/18 patients during surgery, in only one patient before operation and in none post-operatively ($P = 0.025$). Circulating cells were associated with vascular invasion within the primary tumour ($P = 0.032$). No cytokeratin-positive cells were found in blood from ten normal volunteers or four patients undergoing surgery for other breast conditions. These results confirm that cancer surgery in humans results in an increase in the shedding of tumour cells into the circulation. The implications for prognosis and practice should be determined by larger prospective studies.

**Keywords:** circulating cell; metastasis; surgery

Blood-borne metastasis is the greatest obstacle to cure in patients with cancer. Metastasis is a complex process by which tumour cells enter blood vessels, circulate and exit the system at distant sites (Hart and Saini, 1992). Trauma to tumours increases both cell shedding and metastasis in animal models (Tyzzer, 1913; Liotta *et al.*, 1976), leading to concern that cancer surgery might have similar effects in humans. Early studies of cell shedding in humans were confounded by major sampling and cell identification errors, and a general appreciation of the inadequacy of contemporary techniques led to a decline in interest in the subject (Salsbury, 1975). In recent years emphasis in metastasis research has shifted to the phenotype of the cancer cell, and a number of enzymatic and adhesion molecules have been implicated in the metastatic process (Fidler, 1991; Hart and Saini, 1992). The importance of the extent of tumour cell shedding into the bloodstream, and the effects of this on cancer surgery have, by contrast, been neglected. The significance of these factors in determining outcome therefore remains unknown.

Monoclonal antibodies against epithelial-restricted epitopes now permit reliable identification of small numbers of carcinoma cells among large numbers of cells of haemopoetic lineage in the bloodstream and bone marrow (Leather *et al.*, 1993; Pantel *et al.*, 1993). Our ability to obtain samples of effluent blood from tumour sites by selective venous cannulation has also improved. Finally, new concerns over the vulnerability of patients have arisen from studies demonstrating the immunosuppressive effects of surgery. These have shown that natural killer (NK) cell function and T cell responses, believed to be central to the immune response to cancer, are suppressed for 7–28 days after moderate to major operations (Lennard *et al.*, 1985; Sedman *et al.*, 1988). The potential induction of metastasis during cancer surgery therefore needs to be reconsidered. An essential part of this study must be to re-evaluate the effect of surgery on the number of tumour cells shed into the bloodstream. We therefore re-examined the phenomenon of intraoperative tumour cell shedding in patients with breast cancer.

### Materials and methods

### *Patients and sampling method*

With the permission of the local Ethics Committee, 18 patients with operable (Union Internationale Contre le Cancer stages I and II) breast cancer were studied while undergoing primary surgical treatment (simple mastectomy or lumpectomy, together with level II axillary clearance). The size, histological grade (Bloom and Richardson) and vascular invasion of the primary tumour and the status of the axillary lymph nodes were analysed by conventional histopathological methods. All cases were reviewed for this purpose by a single pathologist (JN) unaware of the experimental results. Four patients undergoing surgical excision of non-cancerous breast lesions were also studied. Several hours before surgery, a 'drum cartridge' central venous catheter (Abbot Laboratories, UK) was inserted into the brachiocephalic vein ipsilateral to the tumour at the antecubital fossa and the tip manipulated under radiological guidance into the proximal part of the ipsilateral subclavian vein. One 10 ml blood sample was withdrawn immediately before operation, another three during surgery and one 24 h later. The line was flushed with heparinised saline solution between samples and this flush discarded before each sample was taken. Samples were collected into heparinised tubes and processed immediately.

### *Detection of circulating tumour cells*

Blood samples (10 ml) were diluted with phosphate-buffered saline (PBS) and subjected to differential density centrifugation over Ficoll–Hypaque medium (s.g. = 1.077; Pharmacia, Sweden). Mononuclear cells from the interphase were washed twice in PBS and resuspended in 5 ml of Dulbecco's modified Eagle medium (Gibco, Paisley, UK). Ten cytospins per sample were prepared using a cytocentrifuge (Shandon, UK). After 12–24 h air-drying, cytospin slides were fixed in dry acetone for 10 min and incubated with monoclonal mouse anti-human cytokeratin antibody MNF116 (Dako, High Wycombe, UK) at a dilution of 1:100. This recognises a common epitope on cytokeratins 10, 17 and 18 which are expressed in cells of epithelial lineage but absent from haemopoetic and lymphoid cells (Moll *et al.*, 1982). The antibody reaction was developed using the indirect immunoenzyme APAAP (alkaline phosphatase–anti alkaline phosphatase) technique, using levamisole to inhibit any endogenous phosphatase activity. Red cytoplasmic staining of positive cells was assessed by light microscopy.

### *Validation studies*

The sensitivity and specificity of the antibody for breast carcinoma cells in blood samples was evaluated by staining cytospin preparations from human breast cancer cell lines MCF7, ZR75 and MDA/MB231. These cells were uniformly

Correspondence: P McCulloch
Received 25 January 1995; revised 11 July 1995; accepted 28 July 1995

recognised when stained as pure culture preparations. Cells from each of these cell lines were then mixed with 10 ml of heparinised peripheral venous blood from healthy volunteers in 10-fold dilutions from $10^3$ to one cell $ml^{-1}$. Cytospins were prepared from the mononuclear cell layer of the entire 10 ml sample, stained and counted as described. Ten cytospins were prepared to ensure optimum examination of the entire sample. The number of cytokeratin-stained cells in each cytospin was recorded. The experiment was repeated ten times: thus a total of 100 cytospins representing cells from 100 ml of blood were counted at each cell dilution. Blind testing of normal blood samples from ten healthy volunteers was carried out as a negative control.

### *Statistics*

Cell numbers detected were recorded, and cell numbers per ml of blood calculated assuming a Poisson distribution. The low yield and low total cell numbers per sample made exact quantitative analysis inappropriate, so for statistical purposes samples were scored as positive if any cytokeratin-positive cells were observed on cytospin, and negative if none were found. Proportions of positive and negative results were compared for pre-, intra- and post-operative samples and the null hypothesis (that operation makes no difference to proportion of samples positive for cells) tested using McNemar's test. For this purpose the intermediate of the three intraoperative results was used. One-tailed $P$-values were used since the hypothesis that operation reduced cell shedding was not considered. The relationship between individual prognostic indicators and the presence of circulating cells during surgery was analysed by Fisher's exact test.

**Table I** Mean cell recovery from *in vitro* seeding experiments

| *Cell dilution* | *$10^3$ TC $ml^{-1}$* | *$10^2$ TC $ml^{-1}$* | *10 TC $ml^{-1}$* | *1 TC $ml^{-1}$* |
|---|---|---|---|---|
| Mean cell count 10 $ml^{-1}$ | 532 | 46.2 | 3.1 | 0 |
| Mean percentage cell recovery | 5.32 | 4.62 | 3.1 | 0 |
| s.e.mean | 47 | 5.7 | 0.55 | 1 |
| s.d. | 149 | 17.9 | 1.73 | 0.316 |
| CI (95%) ± | 107 | 12.8 | 1.24 | 0.22 |

Each point is the mean of 100 counted cytospins. Error bars show 95% confidence limited (Poisson distribution assumed). TC, tumour cells.

### Results

### *Validation experiments*

The mean numbers of cells detected per ml of blood is shown in Table I. Cells could be consistently identified when mixed with blood from normal volunteers at concentrations of ten cells $ml^{-1}$ upwards. The yield of cells seeded into normal samples ranged from 3.1% to 5.3%, showing a trend to increase with increased initial cell concentration. The standard error varied from 0.47 to 0.55 at densities above one cell $ml^{-1}$, at which level the method became too insensitive for accurate evaluation. No false-positive results were found in blood samples from normal volunteers.

### *Clinical study*

Cells were detected in the blood of only one patient before operation, in 6 of 18 during surgery, and in none 24 h after operation (see Table II; $Z = -1.964$, 1 d.f., $P = 0.025$, one-tailed). In the one patient with detectable cells preoperatively, the number of cells per ml of blood rose 8-fold during surgery (from seven to a peak of 58 cells $ml^{-1}$). Four of the six patients with detectable cells during surgery had cells present in all three intraoperative samples: the other two had cells in two and one sample respectively (see Table IV). Of eight patients with vascular/lymphatic invasion within the primary tumour on histopathological examination, five were positive for circulating cells during surgery, compared with one of ten patients without invasion ($P = 0.032$, Fisher). No relationship was apparent between detectability of circulating cells and T stage, grade, or nodal status (Table III). No cells were detected in the blood of three patients with benign breast disease or one with intraduct carcinoma only.

### Discussion

This study has demonstrated that breast cancer surgery causes shedding of cancer cells into the effluent blood in detectable numbers. The immunohistochemical techniques used were shown to be highly specific and sensitive at cell densities of ten tumour cells $ml^{-1}$ and above. Our observations are therefore not subject to the criticisms that discredited previous studies of this phenomenon. Although the yield of cells was small, the standard error obtained *in vitro* confirmed the reliability of the sampling technique. The process of tumour cell shedding is known to be intermittent, and no accurate estimate of total tumour cell output over time

**Table II** Results of clinical studies

| *Patient* | *Operation* | *No of cells detected* *Before* | *During* | *After* | *T stage* | *N stage* | *Grade* | *VI* |
|---|---|---|---|---|---|---|---|---|
| 1 | WLE and AD | 7 | 58 | 0 | 1 | – | 2 | + |
| 2 | WLE and AD | 0 | 0 | 0 | 1 | – | 1 | – |
| 3 | WLE and AD | 0 | 4 | 0 | 2 | – | 3 | – |
| 4 | WLE | 0 | 3 | 0 | 1 | – | 2 | + |
| 5 | Mx and AD | 0 | 0 | 0 | 2 | – | 2 | – |
| 6 | WLE and AD | 0 | 6 | 0 | 2 | – | 2 | + |
| 7 | WLE and AD | 0 | 0 | 0 | 2 | – | 2 | – |
| 8 | Mx and AD | 0 | 12 | 0 | 3 | + | 2 | + |
| 9 | WLE and AD | 0 | 0 | 0 | 1 | + | 3 | + |
| 10 | WLE and AD | 0 | 0 | 0 | 1 | – | 2 | – |
| 11 | Mx and AD | 0 | 0 | 0 | 2 | + | 2 | – |
| 12 | Mx and AD | 0 | 0 | 0 | 1 | – | 2 | – |
| 13 | WLE and AD | 0 | 0 | 0 | 2 | – | 2 | + |
| 14 | Mx and AD | 0 | 0 | 0 | 1 | – | 2 | – |
| 15 | WLE and AD | 0 | 8 | 0 | 2 | + | 3 | + |
| 16 | WLE and AD | 0 | 0 | 0 | 2 | + | 3 | + |
| 17 | WLE and AD | 0 | 0 | 0 | 2 | – | 1 | – |
| 18 | WLE and AD | 0 | 0 | 0 | 1 | – | 1 | – |

Numbers of tumour cells are the actual number recovered; approximate estimated number of cells per ml = 20 × this, assuming a mean yield of 5%. The highest value of the three intraoperative samples is recorded. Mx, mastectomy; AD, axillary dissection; WLE, wide local excision; VI, vascular invasion.

**Table III** Results of clinical studies

| | *Positive* | *Negative* |
|---|---|---|
| Vascular invasion* | | |
| + | 5 | 3 |
| − | 1 | 9 |
| T stage | | |
| 1 | 2 | 5 |
| 2 | 4 | 7 |
| Nodal status | | |
| + | 3 | 3 |
| − | 3 | 9 |
| Grade (1/2/3) | | |
| 1 | 0 | 3 |
| 2 | 4 | 7 |
| 3 | 2 | 2 |

Columns show patients either positive or negative for circulating cells during the operation. *$P<0.05$ (Fisher). Numbers are numbers of patients.

**Table IV** Number of tumour cells actually detected in the 18 cases of breast cancer studied

| | *Circulating cells found* | | | | |
|---|---|---|---|---|---|
| *Patient number* | *Before surgery* | *During surgery* | | | *After surgery* |
| | | *1st* | *2nd* | *3rd* | |
| 1 | 7 | 8 | 58 | 5 | 0 |
| 2 | 0 | 0 | 0 | 0 | 0 |
| 3 | 0 | 2 | 4 | 1 | 0 |
| 4 | 0 | 3 | 1 | 1 | 0 |
| 5 | 0 | 0 | 0 | 0 | 0 |
| 6 | 0 | 0 | 0 | 6 | 0 |
| 7 | 0 | 0 | 0 | 0 | 0 |
| 8 | 0 | 8 | 0 | 12 | 0 |
| 9 | 0 | 0 | 0 | 0 | 0 |
| 10 | 0 | 0 | 0 | 0 | 0 |
| 11 | 0 | 0 | 0 | 0 | 0 |
| 12 | 0 | 0 | 0 | 0 | 0 |
| 13 | 0 | 0 | 0 | 0 | 0 |
| 14 | 0 | 0 | 0 | 0 | 0 |
| 15 | 0 | 3 | 1 | 8 | 0 |
| 16 | 0 | 0 | 0 | 0 | 0 |
| 17 | 0 | 0 | 0 | 0 | 0 |
| 18 | 0 | 0 | 0 | 0 | 0 |

The first intraoperative sample was taken within 5 min of the skin incision, and the others at approximately 10 min intervals thereafter.

can be made from these data. These are therefore essentially qualitative results, but they clearly show a difference between the prevalence of tumour cells in the effluent blood before and during cancer surgery. More truly quantitative data may be obtained if the yield of the method can be substantially improved, for example by more extensive blood sampling and semiautomated screening of cytospins. The use of effluent blood samples is essential, since mixed venous blood has already passed through a capillary bed since cell shedding, and this leads to either entrapment or death of most tumour cells (Fidler, 1991).

Two previous studies have reported on intraoperative sampling of effluent venous blood from colon and renal cancers, but neither determined whether surgery had any effect on the rate of cell shedding (Glaves *et al.*, 1988; Leather *et al.*, 1993). Preliminary results using a polymerase chain reaction (PCR) technique to detect circulating tumour cells were similar to our findings, although fewer patients were studied (Brown *et al.*, 1994). The association of cell shedding with vascular invasion found in our patients is in accordance with experimental findings in animal models, in which metastatic potential is related both to tumour vascularity and to the number of clumps of cells released (Liotta *et al.*, 1976). Current surgical opinion assigns little or no importance to cell shedding induced by surgery, but there is little scientific evidence to support this view. Tarin *et al.* (1984) showed massive autotransfusion of tumour cells in ascitic fluid does not necessarily induce metastasis in patients whose cancers were already very advanced. The metabolic, immunological and growth factor environment experienced by cells shed during surgery from a primary tumour without macroscopic metastases may be very different. Tarin's study does not demonstrate that cell numbers are unimportant, merely that other factors are also influential in determining outcome. Studies of the 'no-touch' surgical technique failed to show any survival benefit, but had ample scope for a type II statistical error (failing to detect a real difference due to lack of power). Surgery suppresses cellular immune function and induces expression of growth factors that may stimulate cell division in micrometastases (Lennard *et al.*, 1985; Fisher *et al.*, 1989; Herlyn *et al.*, 1990; McCarthy *et al.*, 1991). Surgery may indirectly induce the expression of integrin IIb/IIIAb on tumour cells by activating the coagulation system and generating thrombin. This platelet-associated integrin may enhance metastatic potential (Wojtukiewicz *et al.*, 1992). Our approach has proved sufficiently reliable to detect tumour cells in blood samples above a threshold level of 10 cells per ml of blood. Other techniques, especially PCR have been investigated for the same purpose, but none to date has shown the specificity of immunohistochemistry in these studies. Avoidance of false-positive findings is essential to the validity of these experiments. It may be necessary to accept a low sensitivity to ensure this, although refinement of the method with semiautomated counting of larger numbers of cytospins may improve this. Further developments in PCR techniques may supplant or complement this approach in the future.

This method, if validated in larger studies, may permit the prognostic significance of effluent tumour cells during surgery to be investigated in the clinical setting. If patients in whom surgery causes increased tumour cell shedding prove to have a poorer prognosis as a result of increased haematogenous metastasis, discarded concepts of intraoperative vascular isolation and 'no touch' techniques may need to be re-evaluated. The significance of cell shedding rates in other situations such as mammography and needle biopsy may also require consideration. Larger studies to permit detailed subgroup analysis, follow-up and evaluation of prognostic significance are now required to determine the clinical significance of these observations.

**Acknowledgements**
This work was supported by grant number 1615 from the Research and Development Fund, University of Liverpool. Mr Choy was supported by a Reseach Fellowship from the Mersey Regional Health Authority. Pathological review for grading and vascular invasion was carried out by Dr John Nash, Reader in Pathology, University of Liverpool. We would like to thank Professor GH Whitehouse for assisting with placement of the subclavian catheters and Professor S Leinster for allowing us to study his patients and for reviewing the manuscript.

**References**

BROWN DC, BIRNIE G, PURUSHOTHAM AD AND GEORGE WD. (1994). Detection of intraoperative shedding of tumour cells in breast cancer by reverse transcription and polymerase chain reaction. *Br. J. Surg.*, **81,** 749.

FIDLER IJ. (1991). The biology of human cancer metastasis. *Acta Oncol.*, **30,** 669–675.

FISHER B, SAFFER E, GUNDUZ N, COYLE J AND RUDOCK C. (1990). Serum growth factor following primary tumour removal and the inhibition of its production by preoperative therapy. *Prog. Clin. Biol. Res.*, **354A,** 47–60.

GLAVES D, HUBEN RP AND WEISS L. (1988). Haematogenous dissemination of cells from human renal adenocarcinomas. *Br. J. Cancer*, **54**, 32-35.

HART I AND SAINI A. (1992). Biology of tumour metastasis. *Lancet*, **339**, 1453-1457.

HERLYN M, KATH R, WILLIAMS N, VALYI NAGI I AND RODECK U. (1990). Growth regulatory factors for normal, premalignant and malignant human cells *in vitro*. *Adv. Cancer Res.*, **54**, 213-234.

LEATHER AJM, GALLEGOS NC, KOCJAN G, SAVAGE F, SMALES CS, HU W, BOULOS PB, NORTHOVER JM AND PHILLIPS RK. (1993). Detection and enumeration of circulating tumour cells in colorectal cancer. *Br. J. Surg.*, **80**, 777-780.

LENNARD TWJ, SHENTON BK, BORZOTTA A, DONNELLY PK, WHITE M, GERRIE LM, PROUD G AND TAYLOR RMR. (1985). The influence of surgical operations on components of the human immune system. *Br. J. Surg.*, **72**, 771-776.

LIOTTA L, KLEINERMAN J AND SAIDEL GM. (1976). The significance of hematogenous tumour cell clumps in the metastatic process. *Cancer Res.*, **36**, 889-894.

McCARTHY JB, SKUBITZ APN, IIDA J, MOORADIAN DL, WILKE MS AND FURCHT LT. (1991). Tumour cell adhesive mechanisms and their relationship to metastasis. *Semin. Cancer Biol.*, **2**, 155-167.

MOLL R, FRANKE WW AND SCHILLER DL. (1982). The catalog of human cytokeratins: patterns of expression in normal epithelia, tumours and cultured cells. *Cell*, **31**, 11-24.

PANTEL K, IZBICKI JR, ANGSTWURM M, BRAUN S, PASSLICK B, KARG O, THETTER O AND RIETHMULLER G. (1993). Immunocytological detection of bone-marrow micrometastasis in operable small cell lung cancer. *Cancer Res.*, **53**, 1027-1031.

SALSBURY AJ. (1975). The significance of the circulating tumour cell. *Cancer Treat. Rev.*, **2**, 55-72.

SEDMAN PC, RAMSDEN CW, BRENNAN TG, GILES GR AND GUILLOU PJ. (1988). Effects of low-dose perioperative interferon on the surgically induced suppression of antitumour immune responses. *Br. J. Surg.*, **75**, 976-981.

TARIN D, PRICE JE, KETTLEWELL MG, SOUTER RG, VASS ACR AND CROSSLEY B. (1984). Mechanisms of human tumour metastasis studied in patients with peritoneovenous shunts. *Cancer Res.*, **44**, 3584-3592.

TYZZER EE. (1913). Factors in the production and growth of tumour metastases. *J. Med. Res.*, **28**, 309-322.

WOJTUKIEWICZ MZ, TANG DG, NELSON KL, WALZ DA, DIGLIO CA AND HONN KV. (1992). Thrombin enhances tumour cell adhesive and metastatic properties via increased alphaIIb/betaIII expression on the cell surface. *Thromb. Res.*, **68**, 233-245.